# Genomic and Functional Analysis of Auxiliary Activity Enzymes in the Maize Anthracnose Pathogen *Colletotrichum graminicola*

**DOI:** 10.3390/microorganisms13092080

**Published:** 2025-09-06

**Authors:** Yafei Wang, Jiaxin Chang, Di Zhang, Jinyao Li, Huawei Luo, Mengjin Liu, Yahui Zhang, Yingjun Cui, Yuehua Geng

**Affiliations:** 1 College of Plant Protection, Henan Agricultural University, Zhengzhou 450002, China; yafeiwang2019@163.com (Y.W.); changjiaxin2104@163.com (J.C.); 16637060304@163.com (D.Z.); lijinyao2412@163.com (J.L.); 17335563163@163.com (H.L.); lmj01129@163.com (M.L.); zyh18003985258@163.com (Y.Z.); 2 Henan Province Plant Protection New Technology Promotion Association, Zhengzhou 450000, China

**Keywords:** *Colletotrichum graminicola*, gene family, auxiliary activity enzymes, expression pattern

## Abstract

*Colletotrichum graminicola*, the causative agent of maize anthracnose leaf blight and stalk rot, severely jeopardizes the healthy development of the maize industry. Auxiliary activity enzymes (AAs), a vital subclass of carbohydrate-active enzymes, act as beneficial accessory proteins for fungi in degrading lignocellulose. This study identified 127 *AA* genes from the genome of *C. graminicola* strain TZ-3 and further analyzed the subcellular localization, conserved motifs, and domains of the proteins encoded by these genes. The *CgAA* genes exhibited significant variations in gene structure, and the structural motifs within their encoded proteins also differed. Subcellular localization analysis revealed that most CgAA proteins were localized in the extracellular space. Moreover, the *CgAA* gene family contained abundant conserved domains, suggesting diverse functionalities and potential roles in various fungal biological processes. Multiple cis-acting regulatory elements related to stress responses and plant hormones were detected in the promoter regions of these genes. This study analyzed the expression patterns of *CgAA* genes during pathogen–host interactions and found that most *CgAA* genes were differentially expressed in the interaction between *C. graminicola* and maize. Coupled with GO functional analysis, it was discovered that *CgAAs* are deeply involved in the interaction between *C. graminicola* and maize, closely associated with the pathogenic mechanisms of the pathogen, and may play crucial roles in the initiation and expansion of fungal infections. These results provide valuable resources for elucidating the functions of *AA* genes and lay the groundwork for sustainable agricultural development through the utilization of *AA* genes in disease control and the breeding of stress-resistant, high-yield crop varieties.

## 1. Introduction

The first line of defense for plants against pathogens is the plant cell wall, primarily composed of polysaccharides such as cellulose and pectin. During the infection process, plant pathogenic fungi secrete cell wall-degrading enzymes to disrupt the plant cell wall, thereby facilitating successful invasion of the host [1,2]. Fungi typically utilize various cellulose-degrading enzymes that synergistically degrade cellulose. The carbohydrate-active enzyme (CAZy) database classifies these enzymes based on sequence similarity into six main classes: glycoside hydrolases (GHs), glycosyltransferases (GTs), polysaccharide lyases (PLs), carbohydrate esterases (CEs), auxiliary activity enzymes (AAs), and carbohydrate-binding modules (CBMs) [2]. Some studies have indicated that members of GHs, PLs, and CEs participate in the degradation of plant cell walls and the pathogenic processes of pathogens [3,4,5,6]. While GHs, PLs, and CEs are well studied, the role of AAs, a key class of redox enzymes, remains poorly understood in fungal pathogenicity.

AAs, a core subclass of CAZymes, focus on redox enzymes that aid in the degradation of recalcitrant polysaccharides via oxidative mechanisms. These enzymes frequently depend on coenzymes to catalyze the generation of reactive oxygen species, synergistically enhancing the accessibility of substrates along with other CAZymes. AAs are indispensable during the process of pathogen infection, as they can disrupt plant cell walls to facilitate successful invasion by pathogens. The AA class is currently divided into nine lignin-degrading enzyme families (AA1–8, AA12) and eight lytic polysaccharide monooxygenase families (AA9–11, AA13–17) [2,7,8]. Members of different AA families have been shown to possess multiple substrate activities, including the oxidative cleavage of crystalline cellulose, hemicellulose, chitin, xylan, xyloglucan, and starch [7,8]. Many AAs in pathogenic fungi possess multiple substrate activities, which are also components of plant cell walls [9,10]. Numerous fungal organisms encode various AA proteins, known for their diversity and abundance in fungal genomes. During the infection of host plants, AAs can not only oxidize and cleave recalcitrant polysaccharides in cell walls to disrupt them but also act as effector molecules involved in the pathogenic process and immune response of plants [11,12,13,14]. Additionally, they can serve as coordinating factors to promote mutualistic symbiosis between different species [15,16].

*Colletotrichum graminicola*, the causative agent of maize anthracnose stem rot, is a worldwide maize disease that can lead to leaf blight, stalk rot, and plant lodging, resulting in yield losses of 30–50% in severe cases [17,18]. Affected by global climate change, this disease has emerged in major maize-producing areas in Northeast and North China in recent years, posing a serious threat to maize production security in China [19]. *C. graminicola* belongs to hemibiotrophic pathogenic fungi, with its infection process initially being biotrophic and gradually transitioning to necrotrophic. During the biotrophic stage, spherical invasion hyphae enveloped by the host plasma membrane develop within living epidermal cells, with hyphae rapidly growing and spreading to kill host cells, followed by the entry of fungi into the necrotrophic stage [20]. Currently, the prevention and control of maize anthracnose primarily rely on benzimidazole chemical agents, which can lead to issues such as pathogen resistance, pesticide residues, and environmental pollution [21]. The increasing occurrence of pathogen resistance undermines the effectiveness of chemical control measures. Given the extensive global cultivation of maize, sustainable solutions are imperative to safeguard food security.

Recently, with the development of molecular biology and genomic technologies, researchers have prompted researchers to delve into the genomic architectures and pathogenicity mechanisms of crop pathogenic fungi [22,23,24]. The completion of the whole-genome sequencing of *C. graminicola* has provided conditions for analyzing genes related to pathogenesis [25]. Currently, research on *AA* gene families in pathogenic fungi is still limited. This study identified and analyzed all *AA*-family genes encoding proteins and their biochemical characteristics by searching the genome of *C. graminicola* TZ-3. Phylogenetic analysis was used to investigate the relationships among *AA* genes, the Gene Structure Display Server (GSDS) for predicting the structural features of *AA* genes, and Multiple Em for Motif Elicitation (MEME) for predicting conserved motifs in AA proteins. We analyzed the response of the *CgAA* gene family during *C. graminicola* infection, and the results indicated that *CgAA* genes may play crucial roles in the pathogenesis of *C. graminicola*. This study expands the available genetic resources of *AA* genes and broadens their potential applications in sustainable agriculture.

## 2. Materials and Methods

### 2.1. Identification and Phylogenetic Analysis of CgAA-Family Genes

To identify *AA*-family genes in *C. graminicola* TZ-3, genome data were obtained from the genome sequencing databases based on previously published information [25]. In the CAZy database, *AA* genes in the *C. graminicola* genome are identified using an e-value cutoff of 1 × 10^−5^. The physicochemical properties of CgAA proteins, including molecular weight, isoelectric point, and GRAVY, were predicted using the online ExPASy website (https://web.expasy.org/protparam/) (accessed on 10 May 2025) [26]. Protein subcellular localization was determined using the PSORT web tool (https://wolfpsort.hgc.jp/) (accessed on 10 May 2025).

### 2.2. Multiple Sequence Alignment and Phylogenetic Analysis

Multiple sequence alignment of AA-family protein sequences from *C. graminicola* TZ-3 was performed using Clustal W with default parameters. Based on the multiple sequence alignment results, a phylogenetic tree was constructed using the maximum likelihood (ML) method in MEGA 7.0. To obtain relatively stable and accurate results, the bootstrap value was set to 1000. The 127 CgAA proteins were grouped according to their clustering in the phylogenetic tree. The evolutionary tree was visually optimized using the Evolview website (http://evolgenius.info/evolview-v2/) (accessed on 20 May 2025) [27].

### 2.3. Gene Structure and Protein Motif Analysis

The GSDS (https://gsds.gao-lab.org/index.php) (accessed on 20 May 2025) was used to predict exons and introns in *CgAA* genes [28]. Conserved motifs in CgAA protein sequences were predicted using the MEME website (http://meme-suite.org) (accessed on 20 May 2025) [29]. To ensure the reliability of the analysis results, the number of motifs in MEME was set to 10, and other parameters were set to default values.

### 2.4. CARE and Gene Ontology (GO) Analysis

The 2000 bp upstream sequences of *CgAA* genes were obtained from the *C. graminicola* TZ-3 genome. CAREs were identified using the PlantCARE online server (http://bioinformatics.psb.ugent.be/webtools/plantcare/html/) (accessed on 20 May 2025) [30]. Visualization was completed using TBtools II v2.142 software [31]. GO functional enrichment analysis was performed using the website (https://www.omicshare.com/tools) (accessed on 20 May 2025) [32].

### 2.5. Analysis of Expression Patterns of CgAA-Family Genes

To study the expression of *CgAA* genes at different infection time points, RNA-seq data (accession number: GSE34632) from *C. graminicola* were collected from previous reports [20]. The selected time points delineate three key infection stages: 24 h post-infection (hpi) marks the initial attachment phase, 36 hpi corresponds to intracellular biotrophic hyphal development, and 60 hpi signifies the emergence of necrotrophic hyphal specialization [20]. A heatmap was generated based on FPKM values using the online website (https://www.omicshare.com/tools) (accessed on 25 May 2025) [32].

### 2.6. RT-qPCR

Transcriptome data validation was performed via RT-qPCR. Primer design utilized Primer Premier 5.0 software. Total RNA isolation from *C. graminicola*-infected maize leaves employed the Tiangen DP441 assay kit (Tiangen, Beijing, China). First-strand cDNA synthesis was conducted using the HiScript III First Strand cDNA Synthesis Kit (Vazyme, Nanjing, China). RT-qPCR amplification utilized the Taq Pro Universal SYBR qPCR Master Mix Kit (Vazyme, China) on an ABI 7500 Real-Time System (Applied Biosystems, Waltham, MA, USA). Gene expression levels, normalized to the *UBQ* reference gene, were quantified using the 2^−∆∆CT^ method [33]. The dataset was statistically analyzed using IBM SPSS Statistics software (v29.0.2.0) and Student’s *t*-test method. Data visualization was created using GraphPad Prism (v9.3).

## 3. Results

### 3.1. Identification and Physicochemical Property Analysis of CgAA Genes

A total of 127 *AA*-family genes (named *CgAA1*–*CgAA127*) were detected in the genome of *C. graminicola* TZ-3. Table 1 lists their detailed biochemical characteristics. The proteins encoded by these genes vary in size, with CgAA76 being the smallest (21.85 kDa) and CgAA106 being the largest (151.92 kDa). These proteins include 90 acidic proteins (PI value < 6.5), 25 basic proteins (PI value > 7.5), and 12 neutral proteins (6.5 < PI value < 7.5). All CgAA proteins belong to 12 different superfamilies, among which 34 CgAA proteins are classified into the AA7 superfamily, 30 into the AA3 superfamily, and 28 into the AA9 superfamily. The grand average of hydropathicity (GRAVY) range of CgAAs is from −0.625 to 0.201, with only seven CgAA proteins having a GRAVY greater than 0, indicating that most CgAA proteins are hydrophilic. Additionally, we predicted the subcellular localization of CgAA proteins and found that 88 CgAA proteins are localized in the extracellular space, 23 in the cytoplasm, 11 in mitochondria, 4 in the plasma membrane, and 1 in the nucleus (Table 1), suggesting that most CgAA proteins exert their biological functions extracellularly.

### 3.2. Phylogenetic Analysis

**Figure 1:**
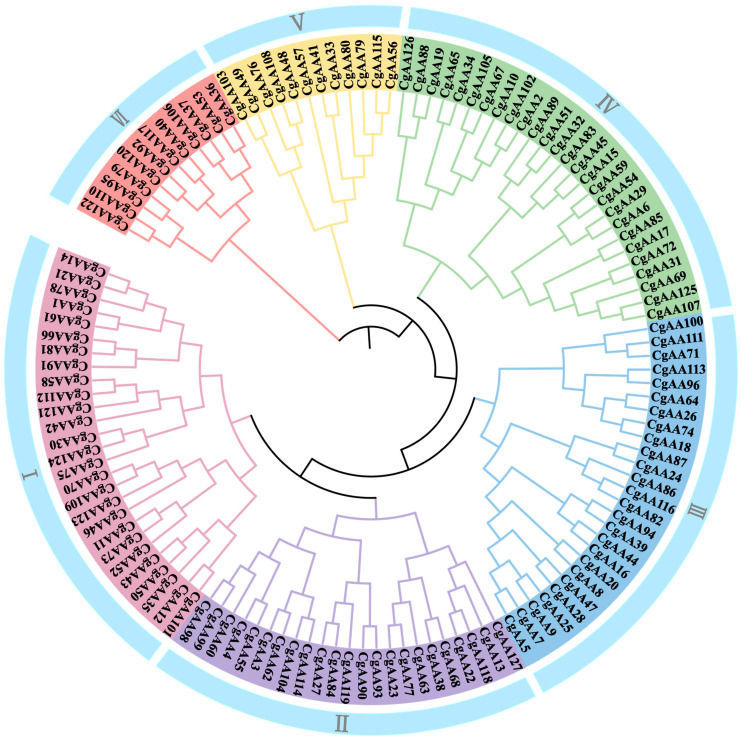
Phylogenetic analysis of AA proteins from *Colletotrichum graminicola*. Note: “Cg” represents *C. graminicola*. The tree reveals that all CgAA proteins can be segregated into six distinct groups (I–VI).

To understand the phylogenetic relationships among CgAA-family proteins, we constructed a phylogenetic tree using the maximum likelihood (ML) method with 127 CgAA protein sequences (Table S1). The phylogenetic tree clusters into six groups, namely I, II, III, IV, V, and VI, with 27, 23, 26, 27, 12, and 12 members, respectively (Figure 1). Groups I and II are phylogenetically close, indicating a relatively tight homologous relationship between these two groups. The distance between Groups I and VI is relatively far, suggesting a distant genetic relationship between them.

### 3.3. Sequence and Structural Analysis

**Figure 2:**
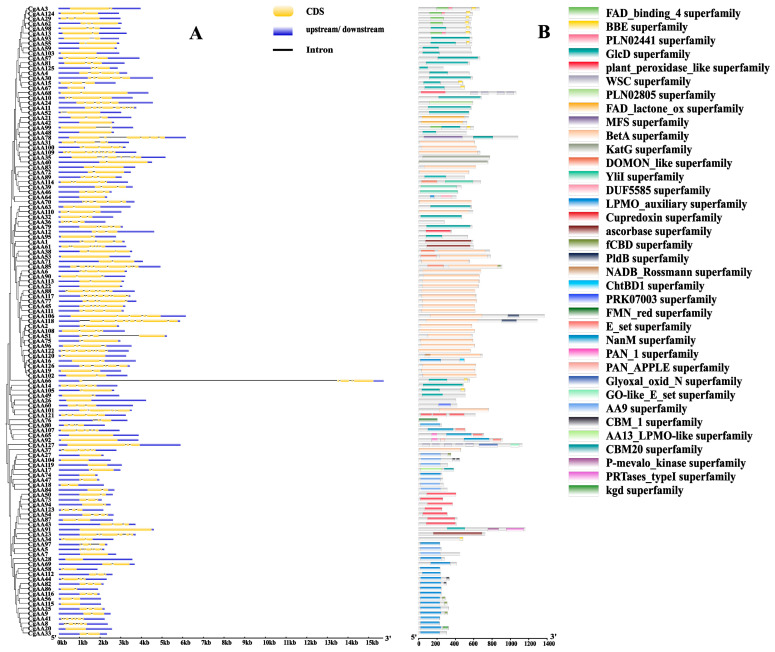
Analysis of gene structures and protein domains of CgAAs. Note: (**A**) Exon–intron organization of *CgAA* genes. Untranslated regions (UTRs) are depicted by blue boxes, exons by yellow boxes, and introns by black lines. (**B**) Domain architecture of CgAA proteins. Distinct color rectangles designate the respective superfamily assignments.

To further elucidate the characteristics of the *AA*-family genes in *C. graminicola* TZ-3, we analyzed the gene structures of *CgAAs* and the conserved domains of their encoded protein sequences (Figure 2, Table S1). As shown in Figure 2A, the 127 *CgAAs* contain zero to nine introns, with the number of introns varying among genes. Among them, *CgAA109* has the most introns (nine), while 19 *CgAAs* have no introns. This indicates that *CgAA* genes may have experienced intron loss or gain events during evolution. The conserved domain analysis of CgAA protein sequences revealed that CgAAs harbor rich domains, with the BetA superfamily domain being the most abundant (34), followed by the GlcD superfamily (28) and LPMO_auxiliary superfamily (21) (Figure 2B).

**Figure 3:**
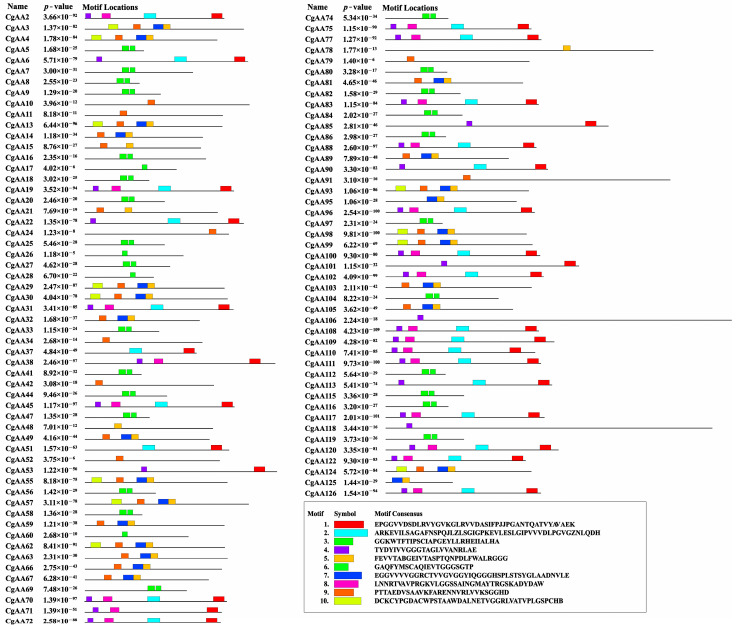
Conserved motif architecture of CgAAs predicted by MEME analysis. Note: Distinctively colored boxes represent diverse conserved motifs varying in length and sequence composition.

To deepen our understanding of the functions of *CgAA* genes, we used the MEME website to predict conserved motifs in CgAA sequences, identifying a total of 10 motifs named motifs 1–10 (Figure 3 and Figure S1). Among them, motif 9 is the most common, present in 33 CgAA proteins. Motifs 1 and 4 are present in 32 CgAA proteins, motif 6 in 31, motif 5 in 28, motifs 2 and 3 in 27, motif 7 in 25, motif 8 in 23, and motif 10 in 12. Twenty-one CgAA proteins contain motifs 1, 2, 4, and 8 simultaneously, while 12 CgAA proteins contain motifs 5, 7, 9, and 10 simultaneously. These motifs play crucial roles in the biological functions of these proteins, and proteins with the same conserved motifs may share similar functions.

### 3.4. Cis-Acting Regulatory Element (CARE) Analysis

**Figure 4:**
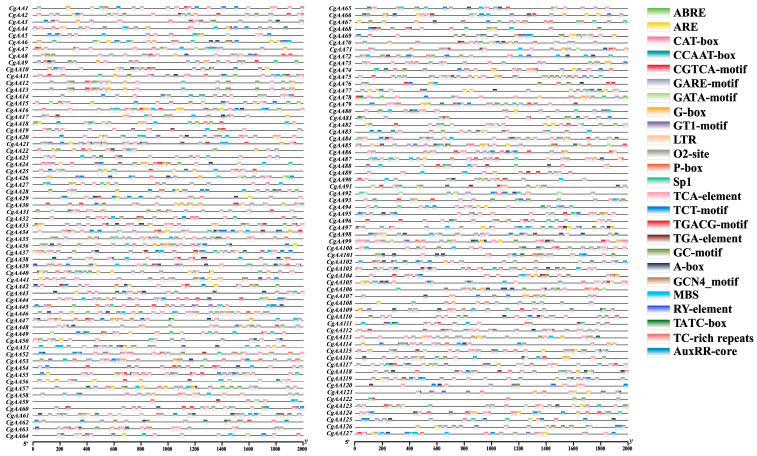
Characterization of cis-acting regulatory elements (CAREs) within the 2 kb promoter regions of *CgAA* genes. Color-coded blocks indicate the distribution and positional arrangement of functionally distinct CAREs.

Analyzing CAREs is crucial for understanding gene regulation [34]. We used the PlantCARE online server to examine the 2000 bp upstream sequences of *CgAA* genes (Table S1) [30]. The results revealed that the promoter regions of *CgAA* genes harbor many cis-regulatory elements related to development, pathogenicity, and stress responses (Figure 4, Table S2). Further analysis showed that the promoter regions of *CgAA* genes contain varying numbers of cis-acting elements, including low-temperature response elements (LTR), drought response elements (MBS), and defense response elements (TC-rich repeats). Hormone-related response elements include MeJA response elements (TGACG-motif and CGTCA-motif), salicylic acid response elements (TCA-element), abscisic acid response elements (ABRE), gibberellin response elements (P-box, GARE-motif, and TATC-box), and auxin response elements (TGA-element and AuxRR-core). Additionally, *CgAA* genes possess CAREs associated with light response (G-Box, Sp1, TCT-motif, GATA-motif, and GT1-motif), zein metabolic regulation (O2-site), anoxic specific inducibility (GC-motif), meristem expression (CAT-box), enhancer-binding protein (CCAAT-box), and seed-specific regulation (RY-element). Among them, MeJA response elements (1064) are the most abundant, followed by light response elements (934) and abscisic acid response elements (486) (Figure S2, Table S2). These findings suggest that *CgAAs* may play important roles in fungal growth, pathogenicity, and responses to various stresses, providing valuable information for understanding the complex regulatory networks constructed by *CgAA* genes in different developmental stages, during pathogenic processes, and under multifactorial stress conditions.

### 3.5. Gene Ontology (GO) Enrichment Analysis

**Figure 5:**
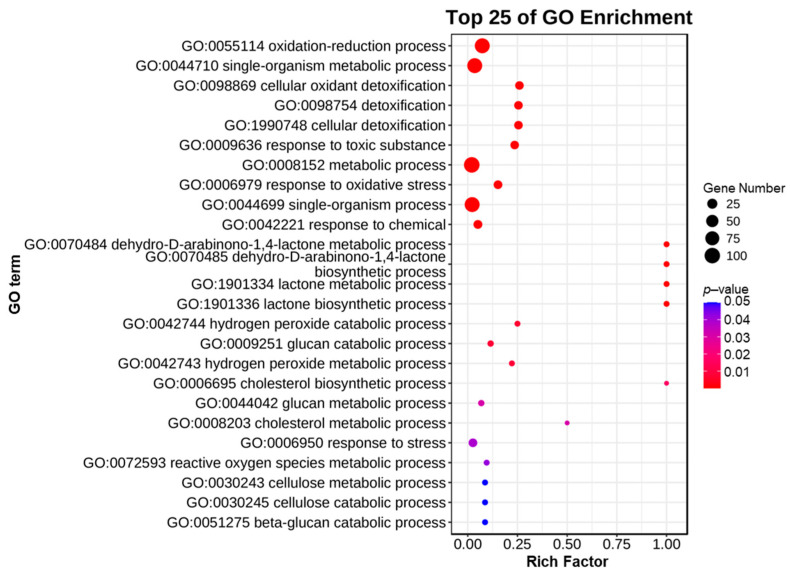
Gene ontology enrichment analysis of *CgAA* genes.

GO enrichment analysis was performed by comparing protein sequences with known functions of species proteins to analyze the functions of these genes. We conducted GO enrichment analysis on 127 *CgAA*-family genes. The results showed that *CgAA* genes are mainly enriched in multiple GO terms, such as oxidation–reduction processes (GO:0055114), single-organism metabolic processes (GO:0044710), cellular oxidation detoxification (GO:0098869), and cellular detoxification (GO:1990748) (Figure 5, Table S3). These results indicate that *CgAA* genes primarily function in oxidation–reduction processes and cellular detoxification.

### 3.6. Response of CgAA-Family Genes During C. graminicola Infection

**Figure 6:**
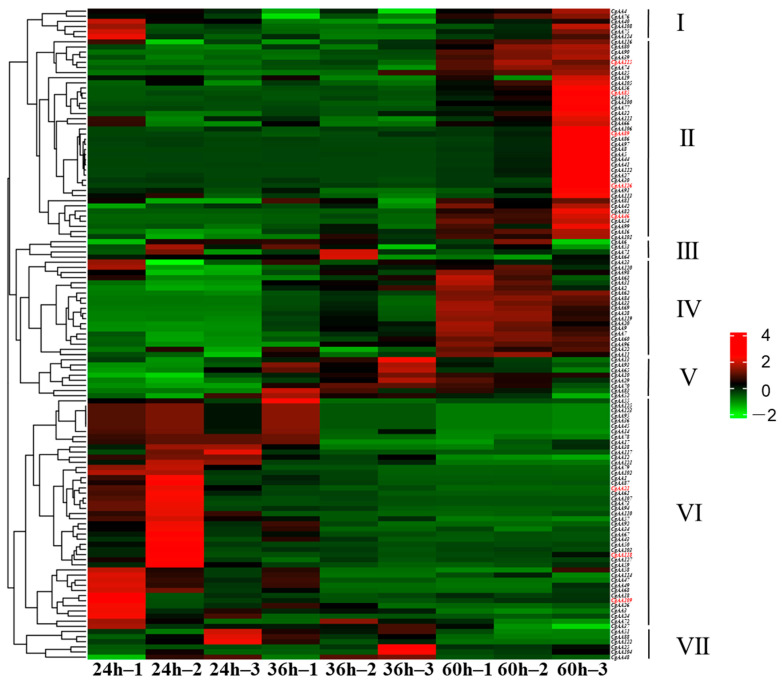
Transcript abundance profiles of *CgAAs* derived from RNA sequencing. Color gradients reflect FPKM values, with red and green hues indicating high and low expression levels, respectively. Genes validated through RT-qPCR are distinguished by red font.

**Figure 7:**
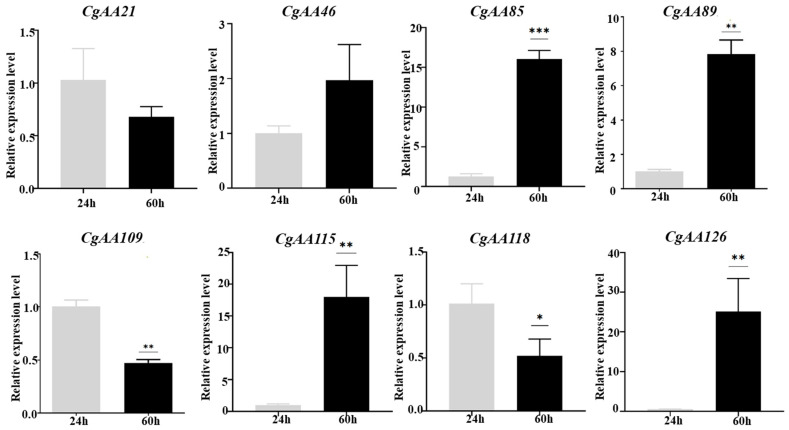
Validation of *CgAA* transcript profiles via RT-qPCR. Histograms depict mean expression levels (±standard error) from three technical replicates. Statistical significance was determined by Student’s *t*-test (n = 3; * *p* < 0.05; ** *p* < 0.01; *** *p* < 0.001).

To analyze *CgAA*-family genes associated with *C. graminicola* infection, we utilized transcriptome data to examine the expression of 127 *CgAA* genes during *C. graminicola* infection of maize. A heatmap was generated based on the FPKM values of 127 *AA*-family genes, showing the expression levels of *CgAAs* at different time points (24 h, 36 h, 60 h) after *C. graminicola* inoculation (Figure 6). Based on the expression profiles of 127 *CgAA*-family genes, they can be clustered into seven categories. These *CgAA*-family genes exhibit significant changes in expression levels during pathogen infection, suggesting their important roles in the process of *C. graminicola* infection. For example, group II contains 39 *CgAA*-family genes, and group IV contains 19 *CgAA*-family genes, both of which have higher transcription levels at 60 h post-inoculation with *C. graminicola*. Group VI contains 45 *CgAA*-family genes, which have higher transcription levels at 24 h post-inoculation. Additionally, we validated the transcriptome data by performing RT-qPCR on eight selected *CgAA* genes (Table S4), confirming the accuracy of the transcriptome data (Figure 7). It should be noted that we selected these representative genes to further validate the significant changes in CgAA genes during the stages of pathogen infection and lesion expansion. Among them, *CgAA21*, *CgAA109*, and *CgAA118* are significantly upregulated at 24 h after infection, while *CgAA46*, *CgAA85*, *CgAA89*, *CgAA115* and *CgAA126* are significantly upregulated at 60 h after infection.

## 4. Discussion

AAs, as an important class of the CAZy database, primarily collaborate with GHs to degrade complex polysaccharides such as lignocellulose through oxidation–reduction reactions, exhibiting significant potential in biodegradation, bioenergy, and disease control [1,2,8]. Belonging to the redox enzyme category, AA-family enzymes disrupt the aromatic polymer structures in lignocellulose through oxidation reactions, providing sites of action for GHs and thus synergistically promoting polysaccharide degradation [35]. AAs secreted by pathogenic fungi participate in the degradation of plant cell walls, and their functions during infection require further investigation [1,2,8]. To explore the diversity of AAs in nature and improve enzyme preparations, it is necessary to further study AA families present in various pathogenic fungi, thereby providing a basis for identifying new targets for disease control.

In this study, we identified 127 *AA*-family genes in *C. graminicola* TZ-3, which encode proteins of diverse sizes, and most CgAA proteins categorized into the lignin-degrading enzyme family. Phylogenetic analysis classified CgAA proteins into different groups, with genetically closely related CgAA proteins located close together on the phylogenetic tree. Subcellular localization analysis revealed that CgAA proteins are widely distributed in five subcellular structures: plasma membrane, cytoplasm, nucleus, mitochondria, and extracellular space. This distribution suggests their potential diverse functions. Notably, most CgAA proteins are localized in the extracellular space, indicating they may be secreted to the outer side of the cell membrane to exert specific functions extracellularly. These extracellular secreted proteins may play a central role in fungal pathogenesis by degrading cell wall components such as cellulose, hemicellulose, and pectin, causing the cell wall structure to disintegrate and creating conditions for pathogen invasion and colonization [36].

Introns play crucial regulatory roles in gene function during evolution, and genes with fewer introns may respond faster to stress factors such as environmental pressures [37,38]. The distribution of introns in microbial genes often exhibits a discrete pattern [39]. In this study, the number of introns varied greatly among the 127 *CgAAs*, with *CgAA109* having 9 introns and 19 *CgAAs* having no introns, suggesting that *CgAA* genes might have undergone intron gain or loss events during evolution. Some CgAA proteins share the same conserved motifs, indicating that these proteins may have similar functions. Additionally, CgAAs possess abundant superfamily domains, combined with the diverse subcellular localization of CgAA proteins, suggesting that CgAAs may have diverse functions and can intervene in fungal biological processes in multiple ways. These rich structural domains also ensure that they can degrade crystalline polysaccharides in lignocellulose through oxidative degradation, providing a carbon source for fungi and disrupting plant cell wall structure [1,9,10].

The presence of CAREs in promoter regions can significantly influence gene function and regulation [40,41]. Studies have shown that many CAREs play crucial roles in multiple gene families [42,43]. CAREs related to development, pathogenicity, and stress responses were found in the promoter regions of *CgAAs*. Stress response elements include low-temperature response elements, drought response elements, and defense response elements. Hormone response elements include MeJA response elements, salicylic acid response elements, abscisic acid response elements, gibberellin response elements, and auxin response elements. Furthermore, *CgAA* genes possess light response elements, zein metabolic regulation response elements, meristem expression response elements, etc. CAREs are key DNA sequences that regulate the expression of *AA*-family genes, and their optimization can improve enzyme production efficiency [40]. These abundant CAREs in the study provide valuable information for understanding the gene functions of *CgAA*s. In-depth exploration of CAREs is essential for uncovering functional genes related to fungal developmental processes, adaptation mechanisms, and pathogenic processes.

The GO analysis of *CgAAs* revealed that they primarily function in oxidation–reduction processes and cellular detoxification. Gene functions are often associated with their expression characteristics [44,45,46]. For example, researchers identified genes related to sexual development and virulence by examining infection-specific transcriptional patterns in the maize pathogen *Cochliobolus heterotrophus* [47]. *CgAA*-family genes exhibit significant differences in expression during *C. graminicola* infection of maize, with some *CgAA* genes having higher transcription levels at 24 h post-infection, while others have higher transcription levels at 60 h. They may play roles at different stages of *C. graminicola* infection. These *CgAA* genes participate in host–microbe interactions and are closely related to the pathogenic mechanisms of pathogens, potentially playing important roles in the establishment and expansion of fungal infections. To elucidate the precise roles of these genes in fungal pathogenesis, further functional characterization of these genes is required. These genes can serve as candidate targets for the development of RNAi-based biopesticides and provide scientific evidence for the control of maize diseases caused by *C. graminicola*.

In recent years, the interaction between *C. graminicola* and maize has been a research focus, with multiple studies providing insights into the pathogenic mechanisms of *C. graminicola* [23,48]. Our study provides comprehensive information on *CgAAs*, which will help ultimately reveal the interaction network between *C. graminicola* and maize. Through genome-wide analysis, we have initially characterized these *CgAAs*. However, these results are primarily based on bioinformatics analyses, and extensive research endeavors are imperative to unravel the functions and molecular mechanisms associated with *CgAA* genes. In addition, *AA* genes play a pivotal role in sustainable agriculture due to their ability to confer resistance against both biotic and abiotic stresses. Through genetic engineering approaches, scientists can enhance or incorporate specific *AA* genes to improve crop stress tolerance, thereby reducing reliance on chemical pesticides. Progress in RNA interference biopesticides and genetic engineering methodologies has established the technical foundation for *AA* gene utilization in sustainable agricultural systems. This study provides conditions for future researchers to further explore the new uses of AA-family enzymes in disease prevention and control. It should be noted that some AA proteins may interfere with the host’s immune response, and their safety as biopesticides needs to be evaluated in the future.

## 5. Conclusions

This study conducted a comprehensive and systematic bioinformatics analysis of the *CgAA* gene family, elucidating their physicochemical properties and potential biological functions. We successfully identified 127 *AA* genes in the genome of *C. graminicola* TZ-3 and clarified the expression patterns of *CgAA* genes during *C. graminicola* infection of maize using transcriptome data and quantitative PCR. *CgAA* genes were closely related to the pathogenic mechanisms of pathogens and might play important roles in the initiation and expansion of fungal infections. These analysis of *CgAA* genes also revealed potential targets for fungicide development and maize breeding for enhanced resistance.

## Figures and Tables

**Table 1:** Nomenclature and characteristics of the putative auxiliary activity enzymes (AAs) in *Colletotrichum graminicola*.

Proposed Gene Name	Gene ID	Superfamily	CDS Length (bp)	Protein Length (aa)	Mw (KDa)	PI	GRAVY	Predicted Subcellular Localization
*CgAA1*	EVM0000057	AA1	1782	593	64.93	5.68	−0.345	extracellular, including cell wall
*CgAA2*	EVM0000126	AA3	1743	580	60.9	5.38	−0.083	extracellular, including cell wall
*CgAA3*	EVM0000144	AA7	1986	661	71.92	4.81	−0.029	extracellular, including cell wall
*CgAA4*	EVM0000166	AA7	1656	551	58.32	5.54	−0.053	extracellular, including cell wall
*CgAA5*	EVM0000238	AA9	741	246	25.91	8.12	−0.008	extracellular, including cell wall
*CgAA6*	EVM0000361	AA3	2040	679	72.90	5.81	−0.177	extracellular, including cell wall
*CgAA7*	EVM0000376	AA9	1353	450	45.87	4.13	−0.437	extracellular, including cell wall
*CgAA8*	EVM0000455	AA9	687	228	24.59	7.68	−0.241	extracellular, including cell wall
*CgAA9*	EVM0000611	AA9	951	316	32.77	8.81	−0.211	extracellular, including cell wall
*CgAA10*	EVM0000699	AA7	2058	685	74.88	6.55	−0.232	cytosol
*CgAA11*	EVM0000709	AA7	1725	574	62.16	8.75	−0.143	mitochondrion
*CgAA12*	EVM0000712	AA2	1086	361	40.36	8.77	−0.547	mitochondrion
*CgAA13*	EVM0000957	AA7	1719	572	61.55	6.16	−0.062	extracellular, including cell wall
*CgAA14*	EVM0001083	AA7	1476	491	55.45	6.23	−0.33	mitochondrion
*CgAA15*	EVM0001273	AA7	1452	483	52.84	5.34	−0.176	extracellular, including cell wall
*CgAA16*	EVM0001281	AA9	1515	504	51.71	5.21	−0.167	extracellular, including cell wall
*CgAA17*	EVM0001353	AA13	1149	382	40.65	5.65	−0.17	extracellular, including cell wall
*CgAA18*	EVM0001466	AA9	807	268	28.44	5.58	−0.111	extracellular, including cell wall
*CgAA19*	EVM0001491	AA3	1845	614	65.27	6.87	−0.058	extracellular, including cell wall
*CgAA20*	EVM0001559	AA9	990	329	34.12	9.35	−0.43	extracellular, including cell wall
*CgAA21*	EVM0001679	AA7	1644	547	61.99	5.96	−0.325	cytosol
*CgAA22*	EVM0001699	AA3	1965	654	70.80	7.21	−0.24	extracellular, including cell wall
*CgAA23*	EVM0001906	AA1	2175	724	80.10	5.64	−0.408	plasma membrane
*CgAA24*	EVM0002162	AA4	1782	593	63.89	6.08	−0.154	mitochondrion
*CgAA25*	EVM0002332	AA9	990	329	34.92	8.83	−0.368	extracellular, including cell wall
*CgAA26*	EVM0002385	AA11	1221	406	40.30	6.15	−0.31	extracellular, including cell wall
*CgAA27*	EVM0002467	AA9	1056	351	35.52	6.35	−0.026	extracellular, including cell wall
*CgAA28*	EVM0002775	AA9	855	284	29.82	6.07	−0.382	extracellular, including cell wall
*CgAA29*	EVM0002895	AA7	1728	575	60.88	4.69	−0.039	extracellular, including cell wall
*CgAA30*	EVM0002896	AA7	1767	588	63.74	5.17	−0.146	extracellular, including cell wall
*CgAA31*	EVM0002934	AA3	1839	612	66.89	5.86	−0.427	cytosol
*CgAA32*	EVM0003252	AA7	1422	473	50.24	9.23	0.065	extracellular, including cell wall
*CgAA33*	EVM0003261	AA9	921	306	31.93	5.16	−0.219	extracellular, including cell wall
*CgAA34*	EVM0003287	AA7	1455	484	52.64	6.56	−0.109	extracellular, including cell wall
*CgAA35*	EVM0003431	AA2	2331	776	84.45	5.22	−0.373	extracellular, including cell wall
*CgAA36*	EVM0003471	AA4	843	280	31.33	7.76	−0.444	mitochondrion
*CgAA37*	EVM0003514	AA3	1383	460	50.09	4.82	−0.088	cytosol
*CgAA38*	EVM0003570	AA8	2331	776	82.04	9.37	−0.094	extracellular, including cell wall
*CgAA39*	EVM0003756	AA12	1392	463	49.79	4.67	−0.128	extracellular, including cell wall
*CgAA40*	EVM0003880	AA2	2277	758	83.32	5.87	−0.545	cytosol
*CgAA41*	EVM0004090	AA9	696	231	23.99	8.23	0.046	extracellular, including cell wall
*CgAA42*	EVM0004098	AA7	1581	526	58.30	7.3	−0.306	cytosol
*CgAA43*	EVM0004123	AA2	1236	411	44.54	8.38	−0.367	extracellular, including cell wall
*CgAA44*	EVM0004131	AA9	1008	335	33.27	6.18	−0.049	extracellular, including cell wall
*CgAA45*	EVM0004175	AA3	1833	610	66.16	4.88	−0.134	extracellular, including cell wall
*CgAA46*	EVM0004221	AA12	1287	428	45.35	5.39	−0.168	extracellular, including cell wall
*CgAA47*	EVM0004263	AA9	795	264	28.41	6.64	−0.306	extracellular, including cell wall
*CgAA48*	EVM0004305	AA7	1569	522	58.95	7.82	−0.408	cytosol
*CgAA49*	EVM0004472	AA7	1527	508	53.73	4.69	0.025	extracellular, including cell wall
*CgAA50*	EVM0004531	AA2	1221	406	43.73	4.99	−0.261	extracellular, including cell wall
*CgAA51*	EVM0004535	AA3	1767	588	63.70	5.54	−0.289	cytosol
*CgAA52*	EVM0004566	AA4	1653	550	59.84	6	−0.163	mitochondrion
*CgAA53*	EVM0004677	AA8	2352	783	81.83	6.81	−0.146	extracellular, including cell wall
*CgAA54*	EVM0004740	AA2	939	312	32.10	4.66	−0.065	extracellular, including cell wall
*CgAA55*	EVM0004872	AA7	1746	581	62.47	4.79	−0.106	extracellular, including cell wall
*CgAA56*	EVM0005086	AA9	870	289	31.12	8.01	−0.345	extracellular, including cell wall
*CgAA57*	EVM0005263	AA7	2007	668	72.31	5.58	−0.263	cytosol
*CgAA58*	EVM0005385	AA9	705	234	24.74	6.94	−0.17	extracellular, including cell wall
*CgAA59*	EVM0005502	AA7	1710	569	64.66	6.48	−0.492	cytosol
*CgAA60*	EVM0005674	AA11	1260	419	42.17	7.98	−0.125	extracellular, including cell wall
*CgAA61*	EVM0005728	AA1	1797	598	66.65	5.65	−0.6	extracellular, including cell wall
*CgAA62*	EVM0005808	AA7	1701	566	60.30	5.18	−0.018	extracellular, including cell wall
*CgAA63*	EVM0005820	AA7	1734	577	61.37	5.32	0.201	extracellular, including cell wall
*CgAA64*	EVM0005896	AA11	1242	413	43.45	6.19	−0.144	plasma membrane
*CgAA65*	EVM0006060	AA5	2136	711	76.77	4.78	−0.221	extracellular, including cell wall
*CgAA66*	EVM0006129	AA7	1665	554	59.83	4.72	−0.003	extracellular, including cell wall
*CgAA67*	EVM0006152	AA7	1506	501	53.79	5.66	−0.17	extracellular, including cell wall
*CgAA68*	EVM0006409	AA2	3189	1062	111.38	4.85	−0.186	extracellular, including cell wall
*CgAA69*	EVM0006430	AA9	1239	412	43.08	8.99	−0.168	mitochondrion
*CgAA70*	EVM0006631	AA3	1731	576	62.81	5.66	−0.318	extracellular, including cell wall
*CgAA71*	EVM0006804	AA8	1665	554	58.88	5.27	−0.122	mitochondrion
*CgAA72*	EVM0006864	AA3	1653	550	60.93	6.41	−0.287	cytosol
*CgAA73*	EVM0006882	AA2	792	263	27.92	5.42	−0.06	extracellular, including cell wall
*CgAA74*	EVM0006927	AA9	768	255	26.73	8.77	−0.233	extracellular, including cell wall
*CgAA75*	EVM0007308	AA3	1776	591	62.84	4.91	−0.034	extracellular, including cell wall
*CgAA76*	EVM0007366	AA6	615	204	21.85	5.84	−0.092	mitochondrion
*CgAA77*	EVM0007380	AA3	1896	631	68.74	5.02	−0.124	cytosol
*CgAA78*	EVM0007418	AA7	3255	1084	117.19	7.26	0.189	plasma membrane
*CgAA79*	EVM0007480	AA4	1752	583	65.66	5.65	−0.382	cytosol
*CgAA80*	EVM0007520	AA9	756	251	28.26	6.43	−0.625	extracellular, including cell wall
*CgAA81*	EVM0007574	AA7	1674	557	60.47	5.68	−0.283	cytosol
*CgAA82*	EVM0007697	AA9	918	305	31.00	7.59	−0.057	extracellular, including cell wall
*CgAA83*	EVM0007725	AA3	1875	624	67.63	5.15	−0.106	mitochondrion
*CgAA84*	EVM0007732	AA9	942	313	32.86	5.96	−0.354	extracellular, including cell wall
*CgAA85*	EVM0007737	AA8	2730	909	96.72	8.16	−0.183	extracellular, including cell wall
*CgAA86*	EVM0007831	AA9	741	246	25.65	7.69	−0.048	extracellular, including cell wall
*CgAA87*	EVM0007924	AA2	1266	421	45.04	6.16	−0.208	extracellular, including cell wall
*CgAA88*	EVM0008057	AA3	1848	615	65.53	5.22	−0.026	extracellular, including cell wall
*CgAA89*	EVM0008124	AA7	1509	502	54.46	5.01	−0.09	extracellular, including cell wall
*CgAA90*	EVM0008153	AA3	1989	662	70.41	5.83	−0.128	extracellular, including cell wall
*CgAA91*	EVM0008169	AA7	3486	1161	126.67	7.9	−0.069	cytosol
*CgAA92*	EVM0008372	AA5	2742	913	98.20	8.1	−0.216	extracellular, including cell wall
*CgAA93*	EVM0008382	AA7	1755	584	61.78	5.17	0.042	extracellular, including cell wall
*CgAA94*	EVM0008478	AA2	1110	369	38.97	5.05	−0.111	extracellular, including cell wall
*CgAA95*	EVM0008490	AA7	1605	534	58.43	6.16	−0.181	mitochondrion
*CgAA96*	EVM0008492	AA3	1827	608	65	5.09	−0.065	extracellular, including cell wall
*CgAA97*	EVM0008509	AA9	699	232	24.41	7.67	−0.013	extracellular, including cell wall
*CgAA98*	EVM0008580	AA7	1728	575	61.29	4.98	−0.037	extracellular, including cell wall
*CgAA99*	EVM0008837	AA7	1800	599	64.11	6.08	−0.212	cytosol
*CgAA100*	EVM0009070	AA3	1893	630	68.64	5.21	−0.31	cytosol
*CgAA101*	EVM0009233	AA3	2304	767	83	5.75	−0.171	cytosol
*CgAA102*	EVM0009452	AA3	1884	627	67.62	6.42	−0.16	extracellular, including cell wall
*CgAA103*	EVM0009498	AA7	1740	579	65.67	6.22	−0.493	nucleus
*CgAA104*	EVM0009621	AA9	1347	448	47.15	6.43	−0.234	extracellular, including cell wall
*CgAA105*	EVM0009640	AA7	1518	505	54.52	4.98	0.014	extracellular, including cell wall
*CgAA106*	EVM0009686	AA3	4119	1372	151.92	6.58	−0.351	cytosol
*CgAA107*	EVM0009760	AA5	1521	506	53.98	8.29	−0.18	extracellular, including cell wall
*CgAA108*	EVM0009827	AA3	1827	608	66.51	5.35	−0.183	extracellular, including cell wall
*CgAA109*	EVM0009849	AA3	2010	669	74.5	6.5	−0.45	cytosol
*CgAA110*	EVM0009864	AA3	1782	593	64.55	7.03	−0.141	plasma membrane
*CgAA111*	EVM0009923	AA3	1851	616	66.96	5.58	−0.117	extracellular, including cell wall
*CgAA112*	EVM0010000	AA9	717	238	24.85	7.66	−0.045	extracellular, including cell wall
*CgAA113*	EVM0010002	AA3	1983	660	71.52	5.88	−0.32	extracellular, including cell wall
*CgAA114*	EVM0010164	AA12	2037	678	72.08	5.36	−0.136	extracellular, including cell wall
*CgAA115*	EVM0010284	AA9	936	311	32.71	5.31	−0.261	extracellular, including cell wall
*CgAA116*	EVM0010436	AA9	750	249	27.26	5.39	−0.164	extracellular, including cell wall
*CgAA117*	EVM0010440	AA3	1893	630	67.35	4.84	−0.05	extracellular, including cell wall
*CgAA118*	EVM0010562	AA3	3888	1295	142.46	6.39	−0.394	cytosol
*CgAA119*	EVM0010731	AA9	948	315	32.92	6.38	−0.254	extracellular, including cell wall
*CgAA120*	EVM0010854	AA3	2088	695	75.34	5.6	−0.013	extracellular, including cell wall
*CgAA121*	EVM0010919	AA1	1860	619	66.36	5.34	−0.055	extracellular, including cell wall
*CgAA122*	EVM0011070	AA3	1692	563	61.34	5.71	−0.272	cytosol
*CgAA123*	EVM0011076	AA2	768	255	27.78	4.83	−0.379	extracellular, including cell wall
*CgAA124*	EVM0011162	AA7	1761	586	64.6	4.86	−0.23	extracellular, including cell wall
*CgAA125*	EVM0011582	AA7	813	270	30.7	5.85	−0.486	cytosol
*CgAA126*	EVM0011705	AA3	1875	624	67.44	5.89	−0.199	extracellular, including cell wall
*CgAA127*	EVM0011804	AA5	3393	1130	118.11	4.82	−0.071	extracellular, including cell wall

ID: identity; bp: base pair; aa: amino acid; PI: isoelectric point; Mw: molecular weight; GRAVY: grand average of hydropathicity; KDa: kilodalton.

## Data Availability

The contributions presented in the study are included in the article/Supplementary Materials, and further inquiries can be directed to the corresponding author.

## Supplementary Materials

The following supporting information can be downloaded at: https://www.mdpi.com/article/10.3390/microorganisms13092080/s1, Table S1. *CgAA* genomic, CDS, protein, and promoter sequences. Table S2. Cis-acting regulatory elements are present in the *CgAA* gene promoter regions. Table S3. Gene ontology enrichment analysis of *CgAA* genes. Table S4. The primers used in this study. Figure S1. Sequence logo conserved motif of CgAA proteins. Figure S2. The numbers of predicted CAREs in the *CgAA* promoter regions.

## References

[1] Kubicek C.P., Starr T.L., Glass N.L. (2014). Plant cell wall–degrading enzymes and their secretion in plant-pathogenic fungi. Annu. Rev. Phytopathol..

[2] Lombard V., Ramulu H.G., Drula E., Coutinho P.M., Henrissat B. (2013). The carbohydrate-active enzymes database (CAZy) in 2013. Nucleic Acids Res..

[3] Kang Z., Zingen-Sell I., Buchenauer H. (2005). Infection of wheat spikes by *Fusarium avenaceum* and alterations of cell wall components in the infected tissue. Eur. J. Plant Pathol..

[4] Van Vu B., Itoh K., Nguyen Q.B., Tosa Y., Nakayashiki H. (2012). Cellulases belonging to glycoside hydrolase families 6 and 7 contribute to the virulence of *Magnaporthe oryzae*. Mol. Plant Microbe Interact..

[5] Qin J.X., Li B.H., Zhou S.Y. (2020). A novel glycoside hydrolase 74 xyloglucanase cvgh74a is a virulence factor in *Coniella vitis*. J. Integr. Agric..

[6] Tan X., Hu Y., Jia Y., Hou X., Xu Q., Han C., Wang Q. (2020). A conserved glycoside hydrolase family 7 cellobiohydrolase PsGH7a of *Phytophthora sojae* is required for full virulence on soybean. Front. Microbiol..

[7] Bissaro B., Streit B., Isaksen I., Eijsink V.G.H., Beckham G.T., DuBois J.L., Røhr A.K. (2020). Molecular mechanism of the chitinolytic peroxygenase reaction. Proc. Natl. Acad. Sci. USA.

[8] Chen J., Guo X., Zhu M., Chen C., Li D. (2019). Polysaccharide monooxygenase-catalyzed oxidation of cellulose to glucuronic acid-containing cello-oligosaccharides. Biotechnol. Biofuels.

[9] Polonio Á., Fernández-Ortuño D., de Vicente A., Pérez-García A. (2021). A haustorial-expressed lytic polysaccharide monooxygenase from the cucurbit powdery mildew pathogen *Podosphaera xanthii* contributes to the suppression of chitin-triggered immunity. Mol. Plant Pathol..

[10] Sabbadin F., Urresti S., Henrissat B., Avrova A.O., Welsh L.R.J., Lindley P.J., Csukai M., Squires J.N., Walton P.H., Davies G.J. (2021). Secreted pectin monooxygenases drive plant infection by pathogenic oomycetes. Science.

[11] Li Y., Liu X., Liu M., Wang Y., Zou Y., You Y., Yang L., Hu J., Zhang H., Zheng X. (2020). *Magnaporthe oryzae* auxiliary activity protein MoAa91 functions as chitin–binding protein to induce appressorium formation on artificial inductive surfaces and suppress plant immunity. mBio.

[12] Zarattini M., Corso M., Kadowaki M.A., Monclaro A., Magri S., Milanese I., Jolivet S., de Godoy M.O., Hermans C., Fagard M. (2021). LPMO-oxidized cellulose oligosaccharides evoke immunity in Arabidopsis conferring resistance towards necrotrophic fungus *B. cinerea*. Commun. Biol..

[13] Vandhana T.M., Reyre J.L., Sushmaa D., Berrin J.G., Bissaro B., Madhuprakash J. (2022). On the expansion of biological functions of lytic polysaccharide monooxygenases. New Phytol..

[14] Yue H., Jiang J., Taylor A.J., Leite A.L., Dodds E.D., Du L. (2021). Outer membrane vesicle-mediated codelivery of the antifungal HSAF metabolites and lytic polysaccharide monooxygenase in the predatory lysobacter enzymogenes. ACS Chem. Biol..

[15] Zhang F., Anasontzis G.E., Labourel A., Champion C., Haon M., Kemppainen M., Commun C., Deveau A., Pardo A., Veneault-Fourrey C. (2018). The ectomycorrhizal basidiomycete Laccaria bicolor releases a secreted β-1,4 endoglucanase that plays a key role in symbiosis development. New Phytol..

[16] Green K.A., Becker Y., Tanaka A., Takemoto D., Fitzsimons H.L., Seiler S., Lalucque H., Silar P., Scott B. (2017). SymB and SymC, two membrane associated proteins, are required for Epichloë festucae hyphal cell-cell fusion and maintenance of a mutualistic interaction with Lolium perenne. Mol. Microbiol..

[17] Balmer D., de Papajewski D.V., Planchamp C., Glauser G., Mauch-Mani B. (2013). Induced resistance in maize is based on organ-specific defence responses. Plant J..

[18] Miranda V.J., Porto W.F., Fernandes G.D.R., Pogue R., Nolasco D.O., Araujo A.C.G., Cota L.V., Freitas C.G., Dias S.C., Franco O.L. (2017). Comparative transcriptomic analysis indicates genes associated with local and systemic resistance to *Colletotrichum graminicola* in maize. Sci. Rep..

[19] Duan C.X., Guo C., Yang Z.H., Sun S.L., Zhu Z.D., Wang X.M. (2019). First report of Anthracnose leaf blight of maize caused by *Colletotrichum graminicola* in China. Plant Dis..

[20] O’Connell R.J., Thon M.R., Hacquard S., Amyotte S.G., Kleemann J., Torres M.F., Damm U., Buiate E.A., Epstein L., Alkan N. (2012). Lifestyle transitions in plant pathogenic *Colletotrichum* fungi deciphered by genome and transcriptome analyses. Nat. Genet..

[21] Jiao C., Chen L., Sun C., Jiang Y., Zhai L., Liu H., Shen Z. (2020). Evaluating national ecological risk of agricultural pesticides from 2004 to 2017 in China. Environ. Pollut..

[22] Gong A., Jing Z., Zhang K., Tan Q., Wang G., Liu W. (2020). Bioinformatic analysis and functional characterization of the CFEM proteins in maize anthracnose fungus *Colletotrichum graminicola*. J. Integr. Agric..

[23] Mei J., Li Z., Zhou S., Chen X., Wilson R., Liu W. (2023). Effector secretion and stability in the maize anthracnose pathogen *Colletotrichum graminicola* requires N-linked protein glycosylation and the ER chaperone pathway. New Phytol..

[24] Eisermann I., Weihmann F., Krijger J.J., Kröling C., Hause G., Menzel M., Pienkny S., Kiesow A., Deising H.B., Wirsel S.G.R. (2019). Two genes in a pathogenicity gene cluster encoding secreted proteins are required for appressorial penetration and infection of the maize anthracnose fungus *Colletotrichum graminicola*. Environ. Microbiol..

[25] Shi X., Xia X., Mei J., Gong Z., Zhang J., Xiao Y., Duan C., Liu W. (2023). Genome sequence resource of a *Colletotrichum graminicola* field strain from China. Mol. Plant Microbe Interact..

[26] Wilkins M.R., Gasteiger E., Bairoch A., Sanchez J.C., Williams K.L., Appel R.D., Hochstrasser D.F. (1999). Protein identification and analysis tools in the ExPASy server. Methods Mol. Biol..

[27] Subramanian B., Gao S., Lercher M.J., Hu S., Chen W.H. (2019). Evolview v3: A webserver for visualization, annotation, and management of phylogenetic trees. Nucleic Acids Res..

[28] Hu B., Jin J., Guo A.Y., Zhang H., Luo J., Gao G. (2015). GSDS 2.0: An upgraded gene feature visualization server. Bioinformatics.

[29] Bailey T.L., Boden M., Buske F.A., Frith M., Grant C.E., Clementi L., Ren J., Li W.W., Noble W.S. (2009). MEME SUITE: Tools for motif discovery and searching. Nucleic Acids Res..

[30] Lescot M., Déhais P., Thijs G., Marchal K., Moreau Y., Van de Peer Y., Rouzé P., Rombauts S. (2002). PlantCARE, a database of plant cis-acting regulatory elements and a portal to tools for in silico analysis of promoter sequences. Nucleic Acids Res..

[31] Chen C., Wu Y., Li J., Wang X., Zeng Z., Xu J., Liu Y., Feng J., Chen H., He Y. (2023). TBtools-II: A “one for all, all for one” bioinformatics platform for biological big-data mining. Mol. Plant.

[32] Mu H., Chen J., Huang W., Huang G., Deng M., Hong S., Ai P., Gao C., Zhou H. (2024). OmicShare tools: A zero-code interactive online platform for biological data analysis and visualization. Imeta.

[33] Chen Z., Wang J., Dong D., Lou C., Zhang Y., Wang Y., Yu B., Wang F., Kang G. (2025). Comparative analysis of TaPHT1;9 function using CRISPR-edited mutants, ectopic transgenic plants and their wild types under soil conditions. Plant Soil.

[34] Higo K., Ugawa Y., Iwamoto M., Korenaga T. (1999). Plant cis-acting regulatory DNA elements (PLACE) database: 1999. Nucleic Acids Res..

[35] Chirania P., Holwerda E.K., Giannone R.J., Liang X., Poudel S., Ellis J.C., Bomble Y.J., Hettich R.L., Lynd L.R. (2022). Metaproteomics reveals enzymatic strategies deployed by anaerobic microbiomes to maintain lignocellulose deconstruction at high solids. Nat. Commun..

[36] Wei W., Xu L., Peng H., Zhu W., Tanaka K., Cheng J., Sanguinet K.A., Vandemark G., Chen W. (2022). A fungal extracellular effector inactivates plant polygalacturonase-inhibiting protein. Nat. Commun..

[37] Roy S.W., Penny D. (2007). Patterns of intron loss and gain in plants: Intron loss–dominated evolution and genome-wide comparison of *O. sativa* and *A. thaliana*. Mol. Biol. Evol..

[38] Roy S.W., Gilbert W. (2006). The evolution of spliceosomal introns: Patterns, puzzles and progress. Nat. Rev. Genet..

[39] Xu G., Guo C., Shan H., Kong H. (2012). Divergence of duplicate genes in exon–Intron structure. Proc. Natl. Acad. Sci. USA.

[40] Hernandez-Garcia C.M., Finer J.J. (2014). Identification and validation of promoters and cis-acting regulatory elements. Plant Sci..

[41] Xuan C., Feng M., Li X., Hou Y., Wei C., Zhang X. (2024). Genome-wide identification and expression analysis of chitinase genes in watermelon under abiotic stimuli and *Fusarium oxysporum* infection. Int. J. Mol. Sci..

[42] Wang Y., Huang Q., Chen X., Li H., Chang J., Zhang Y., Wang Y., Shi Y. (2025). Genome-wide Identification and analysis of carbohydrate-binding modules in *Colletotrichum graminicola*. Int. J. Mol. Sci..

[43] Li L., Tang J., Wu A., Fan C., Li H. (2024). Genome-wide identification and functional analysis of the *GUX* gene family in *Eucalyptus grandis*. Int. J. Mol. Sci..

[44] Li W., Wang H., Yu D. (2016). Arabidopsis WRKY transcription factors WRKY12 and WRKY13 oppositely regulate flowering under Short-Day conditions. Mol. Plant.

[45] Yu Y., Liu Z., Wang L., Kim S.G., Seo P.J., Qiao M., Wang N., Li S., Cao X., Park C.M. (2016). WRKY71 accelerates flowering via the direct activation of FLOWERING LOCUS T and LEAFY in *Arabidopsis thaliana*. Plant J..

[46] Zhang C.Q., Xu Y., Lu Y., Yu H.X., Gu M.H., Liu Q.Q. (2011). The WRKY transcription factor OsWRKY78 regulates stem elongation and seed development in rice. Planta.

[47] Yu H., Zhang J., Fan J., Jia W., Lv Y., Pan H., Zhang X. (2024). Infection-specific transcriptional patterns of the maize pathogen *Cochliobolus heterostrophus* unravel genes involved in asexual development and virulence. Mol. Plant Pathol..

[48] Sanz-Martín J.M., Pacheco-Arjona J.R., Bello-Rico V., Vargas W.A., Monod M., Díaz-Mínguez J.M., Thon M.R., Sukno S.A. (2016). A highly conserved metalloprotease effector enhances virulence in the maize anthracnose fungus *Colletotrichum graminicola*. Mol. Plant Pathol..

